# Theory of mind on demand: do we prepare or react?

**DOI:** 10.3389/fpsyg.2026.1693027

**Published:** 2026-02-03

**Authors:** Marion Roth, Stacy Marsella

**Affiliations:** 1 Social CDT, College of Science and Engineering, University of Glasgow, Glasgow, United Kingdom; 2 Khoury College of Computer Sciences, Northeastern University, Boston, MA, United States

**Keywords:** bounded rationality, cognitive biases, heuristics, social cognition, theory of mind

## Abstract

Reasoning about others’ thoughts, emotions, or intentions is a sophisticated human ability. Modelling such a complex phenomenon with limited available resources is a challenging pursuit. This work proposes the hypothesis of demand-driven and reactive ToM in humans as an additional strategy to establish sufficient mental inferences in complex social spaces. The authors consider a perspective of bounded rationality and cognitive costs in conceptualising ToM and understanding how humans form, maintain, and reason with models of others efficiently and effectively. This study presents qualitative data exploring what patterns in human ToM may allow humans to quickly and seemingly effortlessly perform the complex task of inferring other people’s mental states. The results consist of several themes, which point to various heuristics that may be employed in shaping tractable ToM mechanisms. In conclusion, this qualitative approach to understanding ToM efficiency shaped the hypothesis of reactive ToM mechanisms human cognition, which needs to be tested in confirmatory quantitative studies. Study limitations, implications for modelling, and directions for future research are discussed.

## 1 Introduction

As humans, we only have access to our own conscious experience. What is on one person’s mind is inherently inaccessible to any other individual. In psychological research this set of exclusive inner experiences is commonly referred to as *mental states* (Thornton et al., 2023). Theory of Mind (ToM) is the ability to reason about others’ mental states in order to make sense of their actions, predict future behaviour, and respond appropriately (Premack and Woodruff, 1978) by means of what is often termed *mental models* (Jonker et al., 2010). Understanding others’ mental states is a fundamental component of human social interaction (Leslie, 1991).

ToM research was originally focussed on cognitive differences between humans and animals (Premack and Woodruff, 1978) and the development of ToM in children (Wimmer and Perner, 1983). The study and application of ToM research has much expanded and evolved in recent years. Understanding others’ minds informs pedagogical practices (Mulvey et al., 2016), crime interventions (Noel and Westby, 2014), or the modelling of responses in high-stress situations (Yongsatianchot and Marsella, 2019, 2022). With the rise of artificial intelligence (AI), ToM has become more relevant to the development of virtual agents (Yang et al., 2018). For example, mental models are fundamental to effective teamwork (Mathieu et al., 2000): The similarity of mental models across members of a team has been associated with better team performance (Bolstad and Endsley, 1999; Lim and Klein, 2006; Jonker et al., 2010). There is increasing interest in the study and use of mental models in AI research communities (Albrecht and Stone, 2018) and the application of mental models in artificial agents has shifted to the core of a lot of contemporary AI development (Bansal et al., 2019). AI with the ability to reason about humans’ mental states are valued across a range of applications, such as industry (Rocha et al., 2023), health (Garcia-Lopez, 2024), teamwork (Bansal et al., 2019), or safer interaction of robots with humans (Blum et al., 2018).

Psychological researchers are fascinated by the human ability to quickly and effortlessly generate sophisticated inferences about others’ thoughts or feelings with very little information available. The computational study of ToM has created a more specific angle on the phenomenon and a contemporary issue encountered by computer scientists is how brains manage complex tasks with minimal resources. When a social environment becomes more complex than a narrow research environment, the computational inference of mental states turns into an computationally costly task. The resources required to implement sophisticated social reasoning in complex social situations make the computational modelling of ToM in realistic social spaces too costly to realistically implement. The space of possibilities to represent others’ thoughts or feelings is theoretically infinite. Kwisthout and Van Rooij (2020) outline other resource demands and concerns around modelling human cognition with Bayesian approaches. And yet, humans manage this task daily. Neither computer science nor theoretical psychology has specified to date how this issue may be solved in practice, by either a computational model or the brain.

In establishing how a system may successfully reason about others’ mental states, the aspect of processing costs is at the centre of the present considerations. Social inferences are fundamentally complex and require a large amount of resources (Robalino and Robson, 2012). Yet, humans still generate such inferences frequently, quickly, and effortlessly. The human brain appears to employ mechanisms that minimise the cognitive costs spent on ToM while still generating inferences of sufficient quality.

A helpful perspective on this is that of bounded rationality, a concept introduced by Simon (1956). Bounded rationality argues that people’s ability to make decisions is limited by various resources including their knowledge, mental capacity, and time. When faced with these limitations, people will often choose a satisfactory decision over an optimal one. This perspective frames decisions as highly contextual and situation-dependent. In particular, the theory proposes that people will use various heuristics and biases to facilitate decision-making. Applied to ToM, the idea of bounded rationality raises the question of what biases or heuristics are used by people to facilitate satisfactory reasoning about others including what factors in a situation determine what is satisfactory.

Some psychological research proposes more specific ways in which human cognition may address the costs of ToM. Keysar et al. (2003), for example, found that people are often egocentric in their reasoning about others’ mental states, i.e., they employ their own perspectives to predict others’ actions. Phenomena such as egocentrism indicate that there may be explicit heuristics involved in ToM, which may reduce the number of required processes and their associated costs to allow humans to perform ToM quickly and efficiently, but still carefully and in detail when required. Kahneman (2011) illustrates various heuristics linked to other cognitive phenomena. Similar shortcuts may help humans solve their own tractability issues with ToM.

This perspective, influenced by different research fields and applications, points to the question of what specific heuristics may characterise the manifestation of mental models in human ToM to navigate the limited availability of resources in complex social spaces. The aim of the present study is to contribute to the understanding of tractable human mental model generation and use, towards the longer-term goal of modelling the process computationally.

Specifically, the following research question is proposed:

What cognitive heuristics characterise efficient mental model manifestation?

To answer this question, the present research investigates mental models and the reasoning patterns preceding them.

## 2 Methodology

### 2.1 Participants

This study was approved by the ethics board of the Psychology department at the University of Glasgow. With the online platform Prolific 40 participants were recruited for this study. They were from the United Kingdom with at least 10 previously completed studies on Prolific and a 95% approval rate on Prolific. The study was not aimed at specific demographics, which is why those details were not restricted or recorded.

### 2.2 Design

This study is exploratory in nature, asking participants about the mental states of 6 referents: *your mother*, *the ruling party*, *children in general*, *your best friend*, *a bus driver (in general)*, and *the police*, which were presented in random order. Evidence suggests that reasoning about others’ mental states is more accurate with friends than with strangers (Stinson and Ickes, 1992). The referents were therefore selected to tap into different levels of familiarity and specificity, with *your mother* and *your best friend* being close and specific people, *children in general* and *a bus driver (in general)* being unspecified members of social groups, and *the ruling party* and *the police* being collective social groups.

The dependent variable in this study consisted of qualitative recordings. The study collected two types of qualitative data: participants’ examples of the referents’ mental states, and their explanations for each example. Both were measured as open questions.

Thornton et al. (2023) described mental states as the underlying drivers of the social world. In line with the discussion by Simons (1992), the more general term was broken down into more specific elements: *beliefs*, *goals*, *intentions*, *habits*, *preferences*, and *opinions*. Participants were asked to give examples of each mental state for each person of reference. They were then asked to explain why they thought the referent has this mental state.

### 2.3 Materials

**Figure 1:**
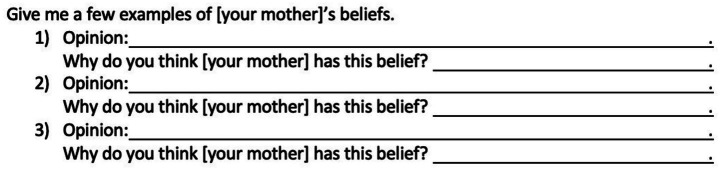
Format of survey questions with the example referent *your mother* and the mental state *belief*.

The questionnaires were administered online, on the survey platform Qualtrics. Questions about different mental states (beliefs, goals, intentions, habits, preferences, and opinions) were all asked in the same format. An example of this for the referent *your mother* and the mental state *belief* is depicted in Figure 1.

### 2.4 Procedure

Participants indicated their interest in this study on Prolific and were redirected to Qualtrics, where they gave informed consent and completed the questionnaire. After completing the study, they received a full debrief and were re-directed back to Prolific where all participants received their payment when data collection was completed.

## 3 Results

In line with the research questions at hand, the results were analysed with the focus on (A) The mechanisms contributing to efficient manifestation of mental models and (B) Inferential patterns guiding the establishment of mental models.

The 6 steps of AMEE Guide No. 131 (Kiger and Varpio, 2020) were followed to achieve a thematic analysis of the data. Thereby, the researcher (1) familiarised themselves with the data, (2) generated initial codes, and (3) searched for overall themes. The themes were (4) reviewed and (5), defined and named, before all results were (6) written up in an overall report. In the following, the four themes that were found and their respective subthemes are presented. Subsequently, the results are discussed as well as how they can inform a computational model.

### 3.1 Data familiarisation

A total of 865 responses was considered in the analysis after exclusion of missing or non-usable data. The average length of answers providing examples of mental states was 4.76 words and the average length of explanations of those mental states was 7.53 words. All responses were actively read several times with the goal of familiarisation and consideration of the research questions.

### 3.2 Codes

**Figure 2:**
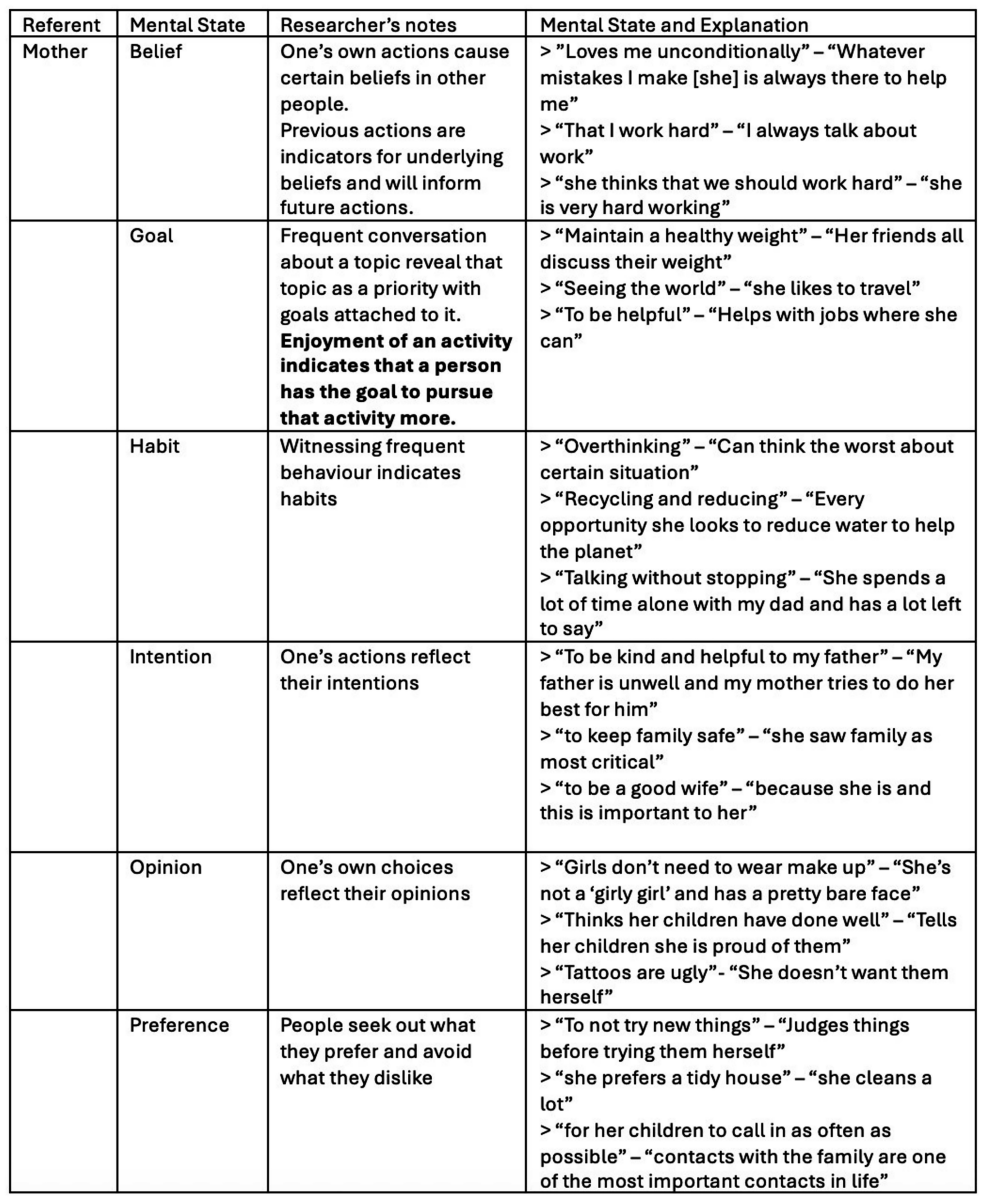
Examples of initial codes for results with supporting responses.

During further reading and consideration of the data, notes were taken on the items of interest. This was done with regard to the mechanisms contributing to efficient manifestation of mental models and the inferential patterns guiding their establishment. Preliminary ideas and potential categories to organise information were collected. Thereby, the referents and mental states were still kept separate and considered individually. Figure 2 shows some examples of these notes.

### 3.3 Initial themes

**Figure 3:**
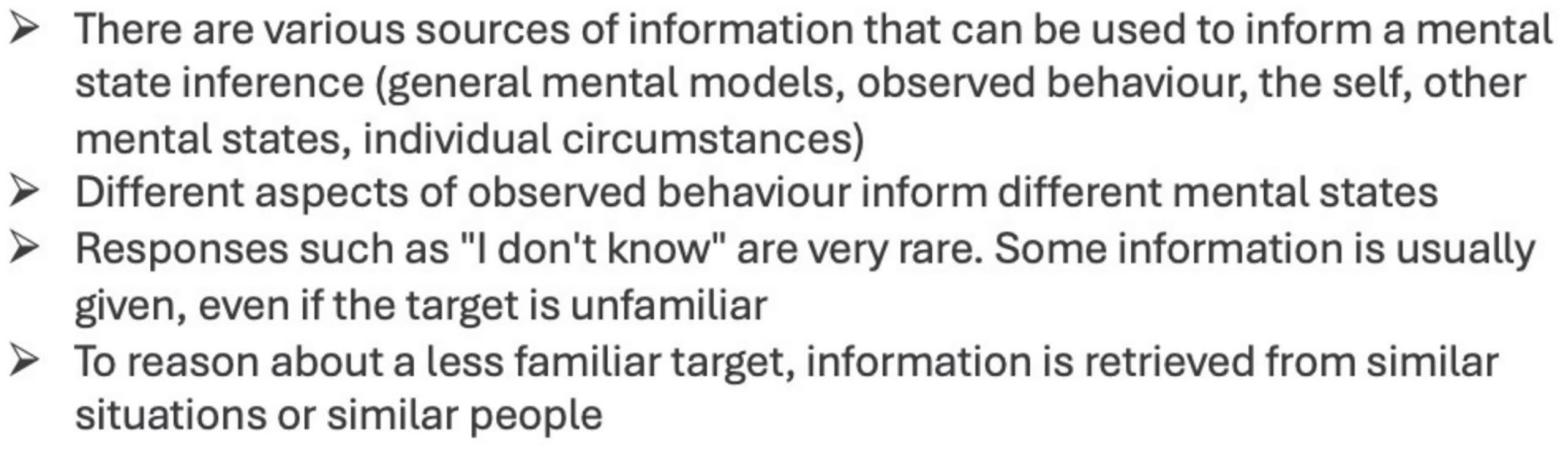
Initial themes connecting various notes and relating back to the research questions.

These notes were reviewed, revised, and thoroughly discussed with other members of the lab. They were then examined for more dense patterns of broader significance, reflecting the data at hand and overarching the various mental states and referents. Mapped together, they informed the establishment of initial themes (Figure 3). Throughout further discussion and evaluation, the final themes were formulated and filled with more detail from all previous notes.

### 3.4 Final themes

#### 3.4.1 Theme 1: *Inferential patterns*

- There are relationships between different mental states. Beliefs, opinions, preferences, goals, intentions, and habits can be inferred from behaviour.

[habit of best friend]” Learning” – “Enrolled to a lot of courses.”

[belief of best friend] “I believe in Green issues” – “Because she campaigns for this”

[intention of mother] “to be a good wife” – “because she is and this is important to her”

[opinion of mother] “Tattoos are ugly”- “She does not want them herself”

[preference of best friend] “Blonde women” – “All his girlfriends were blonde”

- Inferences are often based on other inferences.

[intention of children, in general] “To save money” – “Adults tell them they can use the money to buy what they want and they want that independence”

The data suggests that very different types of mental states were inferred from behaviour and that mental states were also be inferred from other, already inferred, mental states. One participant, for example, inferred the intention of their mother to be “to be a good wife,” “because she is and this is important to her.” To generate this prediction, both her behaviour and her values are referred to. The data suggests that different elements are used flexibly to make sense of others’ mental states and reasons for their behaviour. For example, sometimes preferences can be used to infer intentions, other times values can predict intentions. Depending on the information available, participants used it to express their thoughts about others’ mental states. When people reported back what they reasoned about, only a subset of mental states were mentioned. The results suggest that the activation of mental state representations is highly selective and minimal.

**Figure 4:**
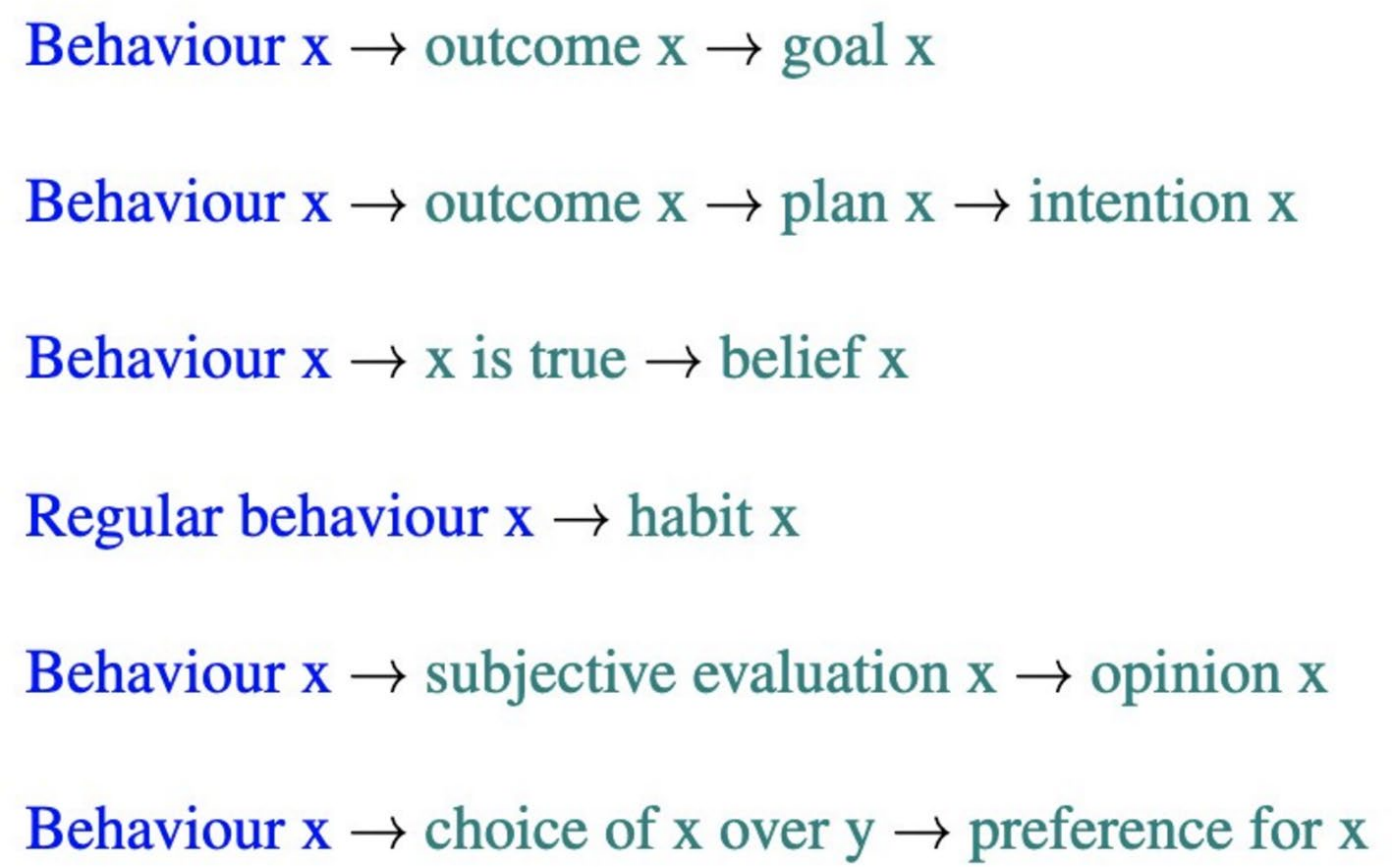
Proposed inferential patterns in ToM, including explanations and inferences.

Figure 4 shows specific inferential patterns that are proposed based on the results above, with inferences highlighted in green and given explanations for those inferences in blue.

#### 3.4.2 Theme 2: *Filling gaps*

- Mental models are based on sampled observations, which are generalised.

[preference of a bus driver, in general] “Drive during the day” – “Evening busses may have some difficult passengers to deal with.”

- When no observations of behaviour are available, mental states can be inferred from a person’s role rather than directly from the individual.

[belief of a bus driver, in general] “public can be rude” – “sure they see it daily.”

If only little information is available about a person, that information has more weight in influencing predictions about them.

[habit of a bus driver, in general] “Walks a lot” – “to [counteract] all the sitting down at work for their shifts.”

- Even fictional information is used to predict others’ mental states.

[preference of a bus driver, in general] “prefers being at home” – “he misses his family”

- When mental states are not known, there is the general assumption that people have the beliefs that are expected of them.

[opinion of children, in general] “They can do what they want” – “Because that’s just children.”

This theme suggests that missing information necessary for reasoning about others is filled in. Different tactics to achieve this were observed, such as generalising from similar situations or a person’s social role. Interestingly, even fictional information was used to fill knowledge gaps. Th present work proposes that at times ToM consists of replacing knowledge gaps with available information that fits into them, even when it is not first-hand information or adopted from a different context.

#### 3.4.3 Theme 3: *Blueprint mental model of “a human”*

This theme captures the phenomenon that people often make specific inferences about behaviour based on a broad assumption of what humans generally do.

- There is the general assumption that people seek out what they prefer and avoid effort or dislike.

[preference of police] “go after easy targets” – “quick wins and boost appearance of solving crime”

- There is the general assumption that behaviour is rooted in an underlying set of values and motivations.

[habit of mother] “Recycling and reducing” – “Every opportunity she looks to reduce water to help the planet.”

Additional to the mechanistic patterns in reasoning about others’ mental states (Theme 1), it appears that there are generally applicable assumptions about people’s behavioural tendencies which help guide the understanding of individual people’s actions. Specifically, regardless of the person, participants seem to assume that others seek out what they prefer and avoid what they dislike or what takes effort. There also seems to be a clear understanding that people generally act based on an underlying set of values and motivations. As part of a general mental model of a human, these assumptions can guide the processing of others’ actions and reduce the resources required to make sense of social situations. This general model may be similar to a blueprint or template, representing how humans generally think, behave, and operate.

#### 3.4.4 Theme 4: *Relationships between in-groups and out-groups*

- Personal approval or disapproval affects the specificity of inferred intentions, leading to a more black-and-white or a more detailed perspective.

[belief of ruling party] “make the rich richer” – “BECAUSE THEY LOOK AFTER THEIR OWN.”

[habit of ruling party] “efficiency” – “lowest unemployment rates in decades”

- There is the general assumption that a person who is similar or different in one way will be similar or different in other ways.

[goal of children, in general] “Stealing candy from the fridge” – “I had this goal as a child.”

[intention of ruling party] “to have fun” – “Because they are elitist and can afford to have fun and break rules”

Finally, in-group or out-group membership appears to affect ToM reasoning. People who are similar in one way are predicted to be similar in other ways as well, whereas people who are different in one way are predicted to also be different in other circumstances. This provides an additional heuristic to reason about others and their mental states. If one can conclude that differences can be generalised to other situations or mental states, unless there is clear contrary information available, this reduces the resources required to infer a mental state. In the following, results will be discussed and insights informing the development of a computational model will be integrated with already existing literature.

## 4 Discussion

### 4.1 Qualitative themes

Recall the research question guiding this work: What cognitive heuristics characterise efficient mental model manifestation? The four themes identified in the qualitative analysis above offer a variety of possible heuristics that may be involved in the efficient manifestation of mental models. In the following, the collected observations of human ToM reasoning will be discussed from a perspective of bounded rationality and efficient cognition with limited resources.

Theme 1, *Inferential patterns* describes sequences extracted from the inference processes recorded in this study. These reasoning sequences represent a narrower mapping of how mental states can be inferred from different aspects of another person’s behaviour. This reduces the number of options to be considered and therefore the cognitive resources required to achieve an inference. These patterns are early propositions and need to be tested and possibly revised in future research. However, eventually, there is the potential to develop such patterns into computational rule sets that can considerably reduce the inference space required in computational ToM by targeting a narrower range of inference possibilities.

The second theme, *Filling gaps*, captures the phenomena of accounting for missing information in reasoning about others’ mental states. Individuals have no direct access to another person’s mental states but even in reasoning and hypothesising about them, helpful information to draw inferences from is often limited. This theme outlines different strategies for how that was navigated by the participants in this study. These strategies can be compared to a phenomenon found in vision science: Each human eye has a blind spot with no photo receptors where the optic nerve meets the eye. However, this lack of visual information is made up for by top-down influences from the brain, regarding what is most likely to be perceived at this point on the visual field (Wandell, 1995). The human brain draws on relevant knowledge to create a complete and consistent experience (Clark, 2013, 2015). This theme proposes that ToM mechanisms operate in a similar way, using already existing, higher-order, and more abstract knowledge to inform lower-level processes and ensure complete representations even when information is missing. Rather than not drawing an inference or acknowledging that there may be too little information available to reliably reason about the another person’s mental states, many participants drew on strategies to establish sufficient evidence for a mental model. For example, this included relying on the own experiences or perspectives, considering what other people in the same role or situation would think or feel, or indeed making up details that were coherent with the other knowledge about that person. ToM is highly characterised by uncertainty (Rusch et al., 2020). However, if an individual considers all the possibilities of another person’s mental states, then the options would be endless, especially if there is little information available. At the same time, humans generally prefer certainty over being left in the dark (Tanovic et al., 2018).

The strategies identified in this theme provide a perspective on how humans may deal with missing information from a bounded rationality perspective: uncertainty is eliminated without spending infinite resources on reasoning about endless possibilities. The findings of this study suggest that different mental models are highly interconnected and dynamic, possibly in order to make up for missing details. It appears that various types of information about the other person are recycled and generalised to different contexts by following the principle that mental states are overall consistent. The harmony between different mental states that this suggests to be part of ToM is consistent with Thagard’s (2002) accounts of coherence and Festinger’s (1957) theory on cognitive dissonance, which are supported by robust bodies of evidence. An important implication for ToM reasoning is that representations of mental states may not be inferred from behaviour independently, but appear to be embedded in a larger network of knowledge about others.

Hebb (Lansner, 2009) refers to this as” cell assembly,” describing a phenomenon of association by simultaneous activation. Similar to how associative memory affects subsequent perception of stimuli (Boettcher et al., 2020), it may be used to provide contextual information in the generation of social inferences. Interestingly, even when the other person is a stranger, the present results suggest that predicted mental states can be incredibly specific. The detail in those representations may be retrieved from templates of social roles and identities, not established in the moment but only accessed when required.

Theme 3, *People in general* proposes general understandings of typical human behaviour as foundations for ToM, independent of the individual. This theme represents the notion that there may be an initial abstract mental model of *a human* applied to every person an individual encounters. Such a basic mental model of how all humans will behave may be less detailed than individual mental models but offer a fundamental heuristic function by providing a template with narrowed-down contents. For example, just like no person is expected to suddenly lift off the ground and float over the floor (model of physical actions), they may be expected to be interested in an object if they are looking at it (model of mental state). Such general principles that may make up a person’s model of people’s mental states in general has the potential to narrow down the possibility of mental state space. From a perspective of bounded rationality, imposing such principles on computational ToM may therefore reduce the amount of information that needs to be processed when a system reasons about others’ mental states. This theme suggests that there are mental states and motivations underlying and guiding all actions, that people behave in a way that will favour and meet their preferences, and that they will avoid discomfort.

And finally, Theme 4, *Relationships between in-groups and out-groups* is concerned with wider social dynamics that influence human ToM beyond the individual that is being reasoned about. Research on ingroup and out-group dynamics is typically focussed on behaviour (Kurzban and Neuberg, 2015). However, the findings here indicate that the understandings of social relations may fundamentally impact more specific and individual attributions of others’ mental states. The theme describes how knowledge about a person’s social environment or place can be utilised to reason about their thoughts, feelings, intentions, or goals. Similar to the previous themes, this can inform inferences and reduce the number of alternatives to be considered. Conceptually, these wider understandings of the social world, how different people and groups relate to each other, and what that means about their mental states, may be a ToM-subset in a wide range of cognitive biases (e.g., Kahneman, 2011). Cognitive biases have the potential to significantly reduce the energy and resources spent on a cognitive task. At the same time, however, these heuristic principles lead to bias in judgement, as they reflect wider social dynamics rather than individual inner experiences. ToM affected by group dynamics and relationships can therefore quickly lead to assumptions without evidence and overly simplistic conclusions.

**Figure 5:**
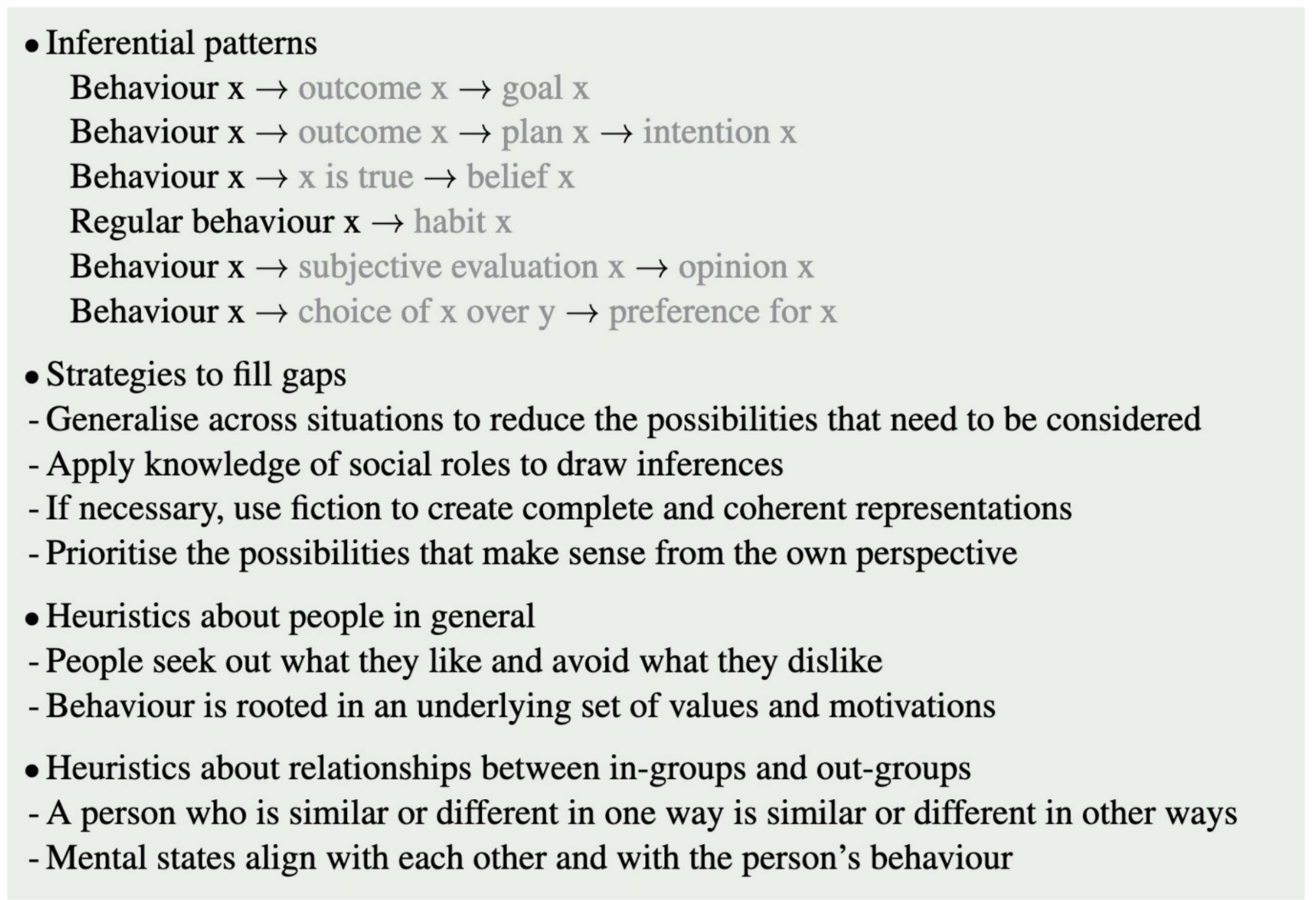
Summary box: overview of heuristics proposed to contribute to tractable ToM in humans.

Figure 5 summarises the heuristics proposed in this work as potential principles that may be tested and revised in future work towards integrating them into computational models.

Collectively, these proposed heuristics suggest more specific ways in which human cognition may realistically complete the rich and manifold task of reasoning about others’ mental states in the complex settings of everyday life with the resources they have available. Kwisthout and Van Rooij (2020) discuss the issue of limited resources and intractability problems in human cognition. The results at hand are qualitative accounts of what may be some strategies to navigate these bounds in human ToM. These strategies can be seen as heuristics in that the objective of their use is to reduce the information space to be considered in social inferences. From a computational point of view, implementing such shortcuts in ToM simulations may reduce the computational resources required to complete such a task. The translation of these qualitative pointers and insights into more tangible computational propositions will be the objective of future studies in line with this work.

### 4.2 Reactive ToM?

Rooted in the themes presented above, this work offers a perspective on the efficient use of available resources and information in the establishment and use of mental models. This work shapes the hypothesis that the formation of mental models can be reactive and on demand, rather than established in preparation and ahead of its requirement. Thus, according to this hypothesis, a person can react with ToM inferences when these are needed in a given situation and pulling together aspects from several different more general models, rather than establishing very specific and tailored representations advance. This reactive and demand-driven perspective suggests a drastic reduction of the number of inferences an individual will generate in the first place. This perspective poses that instead of having mental models readily available, maintained through prior observations and inferences, the demands of a situation may determine whether a person will reason about another person’s mental states. Furthermore, it is proposed here that the requirement to infer another person’s mental state is affected by an overall cost–benefit analysis, in line with the principles of bounded rationality (Simon, 1956): The employment of ToM is constrained by the benefit of a mental model and the cognitive resources available in that moment.

This account of reactive as opposed to proactive ToM shifts the perspective to a more dynamic processing of social stimuli, bounded by both the availability of resources and the value of potential inferences. The notion of how costs and benefits relate to each other and shape an individual’s motivation to employ ToM mechanisms, presents a different angle on efficient ToM use altogether: ToM may come so fast and easily to humans because they may be very selective with reasoning about others’ mental states, based on the perceived benefits and the cognitive energy available to spare. Because different people have very different and personal priorities and preferences, this proposition also aligns with the phenomenon that there is considerable variety in ToM across individuals Conway et al. (2019). It is also consistent with the finding that ToM often fails (Keysar et al., 2003).

The proposition of reactive ToM is particularly in themes 1 and 2. Firstly, the data underlying theme one, *Inferential patterns*, suggests that participants reasoned about the other person’s mental states in response to being asked a specific question, rather than already having the mental model available. Theme 2, *Filling gaps*, offers a perspective on how participants dealt with missing information to reason about others’ mental states. The observation that they so readily used the information they had available to come up with reasonable and coherent conclusions points to an ability to establish mental models reactively and flexibly. It is hypothesised here that the information used for these on-demand mental models may only be retrieved from pre-existing templates, rather than being built from scratch every time a social inference is generated.

From a perspective of bounded rationality and tractability in human ToM processes, a reactive, demanddriven, and availability-based approach to the generation of mental models could explain the flexibility, efficiency, and diversity in ToM observed in previous research (e.g., Gallagher and Fiebich, 2019). The use and reuse of mental model templates and a limitation of ToM the extent that is deemed necessary in a given situation, can drastically reduce the cognitive resources invested in the process.

### 4.3 Limitations and future research

Importantly, however, it needs to be kept in mind that the data at hand is based on an open-ended online survey, which limits the nuances prompted and measured compared to interactional or observational methods. The results at hand are exploratory, and confirmatory studies are needed to assess whether findings can be replicated in a more controlled setting and look at specific aspects in more detail, such as different sources of information underlying a mental model, or the structure and level of detail in general mental models.

Furthermore, future confirmatory work will need to address other factors that may explain the themes at hand. For example, the data suggests that participants could flexibly use already existing knowledge on the spot, which is in line with the hypothesis of reactive ToM. However, the measures used in this work are not designed to rule out the mechanism of proactive and novel generation of mental models as such. It was, for example, not measured how long participants took to answer the questions and what thought processes influenced their final answer. Moreover, it would be interesting to study when more reactive and when more proactive approaches to ToM are employed, to learn more about the possible functions, benefits, and shortcomings of both mechanisms. It may be helpful to research specific measures to identify and record the use of both possibilities, to investigate other factors such as situational and individual differences, cognitive load, or accuracy of inferences.

### 4.4 Conclusion

To conclude, this research has gathered insights from verbal responses on how ToM may be conducted efficiently and within the bounds of limited resources. This work has proposed the hypothesis of reactive ToM mechanisms in human cognition. Several themes have been presented, indicating different heuristics that may contribute to the phenomenon. It has been discussed how these psychological accounts of ToM in a context of resource rationality may be beneficial in improving computational models. Importantly, this research is of exploratory and qualitative nature, and the mechanisms and heuristics proposed here are still to be tested in confirmatory future research. Furthermore, future research required to rule out alternative explanations of the data patterns at hand. A shift in perspective on what strategies characterise human ToM and consideration of the heuristics at hand, however, may provide fundamental benefits for the conceptualisation of ToM and its implementation in computational models. Overall, this work highlights the value of interdisciplinary work to generate new perspectives, test existing hypotheses with different methods, and establish feedback dynamics across fields.

## Data Availability

The raw data supporting the conclusions of this article will be made available by the authors, without undue reservation.

## References

[ref1] Albrecht S. V. Stone P. (2018). Autonomous agents modelling other agents: a comprehensive survey and open problems. Artif. Intell. 258, 66–95. doi: 10.1016/j.artint.2018.01.002

[ref2] Bansal G. Nushi B. Kamar E. Lasecki W. S. Weld D. S. Horvitz E. (2019). “Beyond accuracy: the role of mental models in human-AI team performance” in Proceedings of the AAAI conference on human computation and crowdsourcing, Washington USA: PKP Publishing Services Network, vol. 7, 2–11.

[ref3] Blum C. Winfield A. F. Hafner V. V. (2018). Simulation-based internal models for safer robots. Front. Robot. AI 4:74. doi: 10.3389/frobt.2017.00074

[ref4] Boettcher S. E. Stokes M. G. Nobre A. C. van Ede F. (2020). One thing leads to another: anticipating visual object identity based on associative-memory templates. J. Neurosci. 40, 4010–4020. doi: 10.1523/JNEUROSCI.2751-19.2020 32284338 PMC7219293

[ref5] Bolstad C. A. Endsley M. R. (1999). “Shared mental models and shared displays: an empirical evaluation of team performance” in Proceedings of the human factors and ergonomics society annual meeting, vol. 43 (Los Angeles, CA, USA: SAGE Publications Sage CA), 213–217.

[ref6] Clark A. (2013). Whatever next? Predictive brains, situated agents, and the future of cognitive science. Behav. Brain Sci. 36, 1–73. doi: 10.1017/S0140525X12000477 23663408

[ref7] Clark A. (2015). Surfing uncertainty: prediction, action, and the embodied mind. Oxford, UK: Oxford University Press.

[ref8] Conway J. R. Catmur C. Bird G. (2019). Understanding individual differences in theory of mind via representation of minds, not mental states. Psychon. Bull. Rev. 26, 798–812. doi: 10.3758/s13423-018-1559-x, 30652239 PMC6557866

[ref9] Festinger L. (1957). A theory of cognitive dissonance, vol. 2. Stanford: Stanford University Press.

[ref9001] Gallagher S. Fiebich A. (2019). Being Pluralist About Understanding Others. Knowing Other Minds. 63–78. doi: 10.1093/oso/9780198794400.003.0004

[ref10] Garcia-Lopez A. (2024). “Theory of mind in artificial intelligence applications” in The theory of mind under scrutiny: psychopathology, neuroscience, philosophy of mind and artificial intelligence, Cham: Springer Nature Switzerland, 723–750.

[ref11] Jonker C. M. Riemsdijk M. Vermeulen B. (2010). “Shared mental models” in International workshop on coordination, organizations, institutions, and norms in agent systems (Berlin, Germany: Springer), 132–151.

[ref12] Kahneman D. (2011). Thinking, fast and slow. New York, USA: Macmillan.

[ref13] Keysar B. Lin S. Barr D. J. (2003). Limits on theory of mind use in adults. Cognition 89, 25–41. doi: 10.1016/S0010-0277(03)00064-7 12893123

[ref14] Kiger M. E. Varpio L. (2020). Thematic analysis of qualitative data: AMEE guide no. 131. Med. Teach. 42, 846–854. doi: 10.1080/0142159X.2020.1755030 32356468

[ref15] Kurzban R. Neuberg S. (2015). “Managing ingroup and outgroup relationships” in The handbook of evolutionary psychology (Hoboken, NJ, USA: Wiley Online Library), 653–675.

[ref16] Kwisthout J. Van Rooij I. (2020). Computational resource demands of a predictive Bayesian brain. Comput. Brain Behav. 3, 174–188. doi: 10.1007/s42113-019-00032-3

[ref17] Lansner A. (2009). Associative memory models: from the cell-assembly theory to biophysically detailed cortex simulations. Trends Neurosci. 32, 178–186. doi: 10.1016/j.tins.2008.12.002 19187979

[ref18] Leslie A. M. (1991). “The theory of mind impairment in autism: evidence for a modular mechanism of development?” in Natural theories of mind: evolution, development and simulation of everyday mindreading (Oxford, UK: Basil Blackwell), 63–78.

[ref19] Lim B.-C. Klein K. J. (2006). Team mental models and team performance: a field study of the effects of team mental model similarity and accuracy. J. Org. Behav. Int. J. Ind. Occup. Org. Psychol. Behav. 27, 403–418. doi: 10.1002/job.387

[ref20] Mathieu J. E. Heffner T. S. Goodwin G. F. Salas E. Cannon-Bowers J. A. (2000). The influence of shared mental models on team process and performance. J. Appl. Psychol. 85:273. doi: 10.1037/0021-9010.85.2.273 10783543

[ref21] Mulvey K. L. Rizzo M. T. Killen M. (2016). Challenging gender stereotypes: theory of mind and peer group dynamics. Dev. Sci. 19, 999–1010. doi: 10.1111/desc.12345 26395753 PMC4808471

[ref22] Noel K. K. Westby C. (2014). Applying theory of mind concepts when designing interventions targeting social cognition among youth offenders. Top. Lang. Disord. 34, 344–361. doi: 10.1097/TLD.0000000000000036

[ref23] Premack D. Woodruff G. (1978). Does the chimpanzee have a theory of mind? Behav. Brain Sci. 1, 515–526. doi: 10.1017/S0140525X00076512

[ref24] Robalino N. Robson A. (2012). The economic approach to ‘theory of mind’. Philos. Trans. R. Soc. B Biol. Sci. 367, 2224–2233. doi: 10.1098/rstb.2012.0124 PMC3385689 22734065

[ref25] Rocha M. da Silva H. H. Morales A. S. Sarkadi S. Panisson A. R. (2023). “Applying theory of mind to multi-agent systems: a systematic review” in Brazilian conference on intelligent systems (Berlin: Springer), 367–381.

[ref26] Rusch T. Steixner-Kumar S. Doshi P. Spezio M. Glascher J. (2020). Theory of mind and¨ decision science: towards a typology of tasks and computational models. Neuropsychologia 146:107488. doi: 10.1016/j.neuropsychologia.2020.107488 32407906

[ref27] Simon H. A. (1956). Rational choice and the structure of the environment. Psychol. Rev. 63:129. doi: 10.1037/h0042769 13310708

[ref28] Simons K. W. (1992). Rethinking mental states. BUL Rev 72:463.

[ref29] Stinson L. Ickes W. (1992). Empathic accuracy in the interactions of male friends versus male strangers. J. Pers. Soc. Psychol. 62:787. doi: 10.1037//0022-3514.62.5.787 1593418

[ref30] Tanovic E. Gee D. G. Joormann J. (2018). Intolerance of uncertainty: neural and psychophysiological correlates of the perception of uncertainty as threatening. Clin. Psychol. Rev. 60, 87–99. doi: 10.1016/j.cpr.2018.01.001 29331446

[ref31] Thagard P. (2002). Coherence in thought and action. Cambridge, MA, USA: MIT Press.

[ref32] Thornton M. A. Rmus M. Tamir D. Tamir D. I. (2023). Transition dynamics shape mental state concepts. Journal of Experimental Psychology: General 152, 2804–2829. doi: 10.1037/xge0001405 PMC10593093 37104795

[ref33] Wandell B. A. (1995). Foundations of vision. Sunderland, MA, USA: Sinauer Associates.

[ref34] Wimmer H. Perner J. (1983). Beliefs about beliefs: representation and constraircing function of wrong beliefs in young children’s understanding of deception. Cognition 13, 103–128. doi: 10.1016/0010-0277(83)90004-5 6681741

[ref35] Yang G.-Z. Bellingham J. Dupont P. E. Fischer P. Floridi L. Full R. . (2018). The grand challenges of science robotics. Sci. Robot. 3, 1–14. doi: 10.1126/scirobotics.aar7650 33141701

[ref36] Yongsatianchot N. Marsella S. (2019). “Modeling human decision-making during hurricanes: from model to data collection to prediction” in AAMAS conference proceedings (Alexandria: National Science Foundation). doi: 10.1109/TAFFC.2022.3173812

[ref37] Yongsatianchot N. Marsella S. (2022). A computational model of coping and decision making in high-stress, uncertain situations: an application to hurricane evacuation decisions. IEEE Trans. Affect. Comput. 14, 2539–2556.

